# Structure-Function Analysis of Nucleolin and ErbB Receptors Interactions

**DOI:** 10.1371/journal.pone.0006128

**Published:** 2009-07-03

**Authors:** Keren Farin, Ayelet Di Segni, Adam Mor, Ronit Pinkas-Kramarski

**Affiliations:** Department of Neurobiology, Tel-Aviv University, Ramat-Aviv, Israel; Universidade Federal do Rio de Janeiro (UFRJ), Instituto de Biofísica da UFRJ, Brazil

## Abstract

**Background:**

The ErbB receptor tyrosine kinases and nucleolin are major contributors to malignant transformation. Recently we have found that cell-surface ErbB receptors interact with nucleolin via their cytoplasmic tail. Overexpression of ErbB1 and nucleolin leads to receptor phosphorylation, dimerization and anchorage independent growth.

**Methodology/Principal Findings:**

In the present study we explored the regions of nucleolin and ErbB responsible for their interaction. Using mutational analyses, we addressed the structure–function relationship of the interaction between ErbB1 and nucleolin. We identified the ErbB1 nuclear localization domain as nucleolin interacting region. This region is important for nucleolin-associated receptor activation. Notably, though the tyrosine kinase domain is important for nucleolin-associated receptor activation, it is not involved in nucleolin/ErbB interactions. In addition, we demonstrated that the 212 c-terminal portion of nucleolin is imperative for the interaction with ErbB1 and ErbB4. This region of nucleolin is sufficient to induce ErbB1 dimerization, phosphorylation and growth in soft agar.

**Conclusions/Significance:**

The oncogenic potential of ErbB depends on receptor levels and activation. Nucleolin affects ErbB dimerization and activation leading to enhanced cell growth. The C-terminal region of nucleolin and the ErbB1 NLS-domain mediate this interaction. Moreover, when the C-terminal 212 amino acids region of nucleolin is expressed with ErbB1, it can enhance anchorage independent cell growth. Taken together these results offer new insight into the role of ErbB1 and nucleolin interaction in malignant cells.

## Introduction

The ErbB subfamily of receptor tyrosine kinases (RTK) consists of four receptors: epidermal growth factor receptor (EGFR,HER1,ErbB1), HER2 (ErbB2,neu), HER3 (ErbB3) and HER4 (ErbB4) [1]–[7]. These receptors are mediators of growth signals and thus determine cell fate. They are cell surface allosteric enzymes consisting of a single transmembrane domain that separates the intracellular kinase domain from the extracellular ligand-binding domain. Ligand binding to the ectodomain results in allosteric alterations leading to receptor homo- or hetero-dimerization, kinase activation, and trans-autophosphorylation [8]–[10]. Subsequent tyrosine phosphorylation on residues within the carboxyl terminal tail of the receptors enables the recruitment and activation of signaling effectors containing Src homology 2 (SH2) domain and phosphotyrosine binding (PTB) domain that will lead to the initiation of various signaling cascades [11]–[13]. The ErbB receptors are expressed in various tissues of epithelial, mesenchymal and neuronal origin. Under normal physiological conditions, activation of the ErbB receptors is controlled by the spatial and temporal expression of their ligands, which are members of the EGF family of growth factors [14]. Abnormal function of the ErbB receptors and their ligands is involved in human cancer and already serve as target for several cancer drugs [15].

Nucleolin is a ubiquitous expressed acidic phosphoprotein of exponentially growing cells. It is involved mainly in the synthesis and maturation of ribosomes [16]. Nucleolin is a nonhistone nucleolar phosphoprotein, present mainly at the dense fibrillar and granular regions of the nucleolus [17], [18]. In nondividing cells, degraded forms of various molecular sizes are predominantly expressed due to auto degradation [19]. Hence, the protein appears to be involved in essential aspects of transcriptional regulation, cell proliferation, and growth [20], [21]. Recently, it was demonstrated that nucleolin is a multifunctional shuttling protein present in nucleus, cytoplasm, and on the surface of some types of cells [16], [22], [23]. It was also demonstrated that inhibition of cell-surface nucleolin suppresses tumor growth and angiogenesis [24]. Inhibition of nucleolin activity results in cell growth inhibition [25]. In addition, it was demonstrated in several studies that AS1411 (or GROA), a quadruplex-forming oligonucleotide aptamer, targets nucleolin and inhibits cancer cells growth [26], [27]. Also, it was demonstrated that inhibition of cell-surface expressed nucleolin, by HB-19 pseudo peptide, suppresses tumor growth and angiogenesis [24]. These effects of cell-surface nucleolin inhibition were demonstrated on breast, prostate and glioma cell lines which also express high levels of ErbB receptors. In our most recent study we identified nucleolin as an ErbB receptors interacting protein [28]. This interaction on the cell surface, leads to receptor dimerization and activation as well as to colonies growth on soft agar [28]. We therefore suggested that the cross talk between nucleolin and ErbB proteins may be related to tumor growth.

Nucleolin protein contains several functional domains that mediate its functions [29]. The N-terminal part contains multiple phosphorylation sites and is rich in acidic amino acids. The central part of nucleolin includes four RNA binding domains (RBD) and the C-terminal part contains glycine and arginine rich domain (termed RGG or GAR domain) [16], [30], [31]. In the present study we demonstrate that ErbB1 juxtamembrane region (the nuclear localization sequence NLS), which is important for ErbB1 kinase activation [32], is also important for nucleolin-ErbB1 interaction and nucleolin-induced ErbB1 dimerization and activation. In addition, we further demonstrate that the 212 C-terminal amino acids of nucleolin (RBD4 and GAR domains) are sufficient for cell surface localization and for ErbB1 interaction and activation. Moreover, the 212 C-terminal amino acids of nucleolin when expressed with ErbB1, co localize to the cell membrane and enhance colonies formation in soft agar.

## Results

### Analysis of ErbB1 domains responsible for nucleolin/ErbB1 interaction

**Figure 1:**
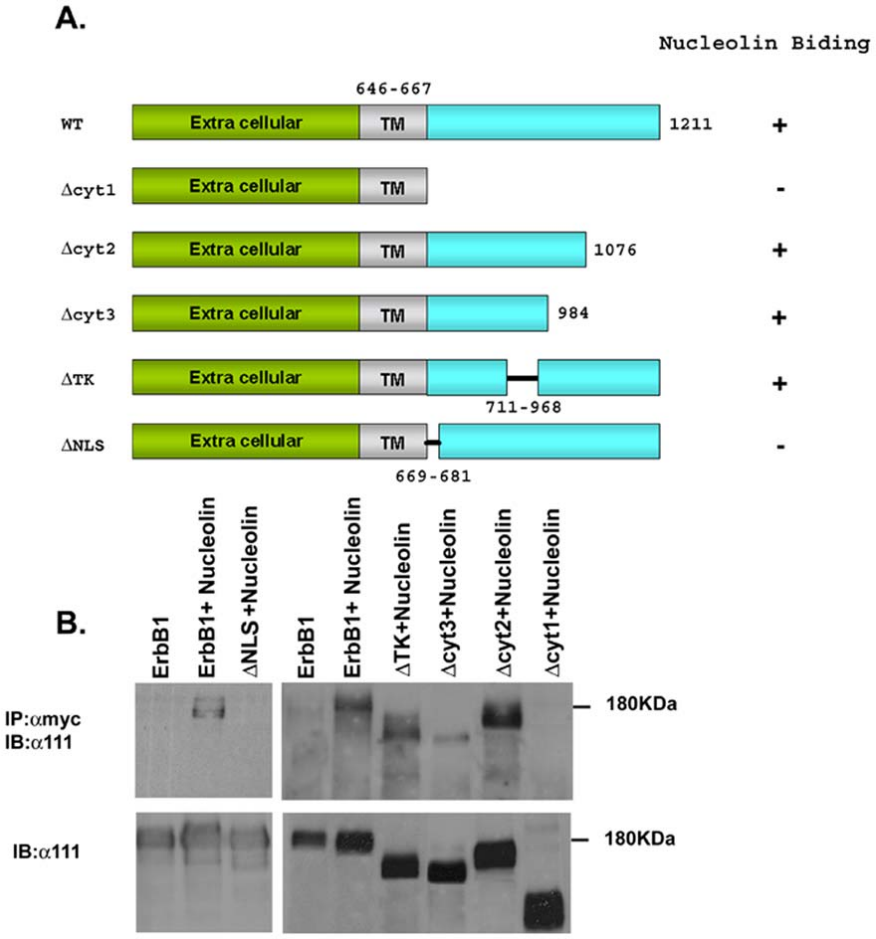
Analysis of the ErbB1 regions important for ErbB1 nucleolin interaction. (A) Schematic presentation of ErbB1 deletion mutants. (B) Cells were co-transfected with pEGFP-nucleolin-Myc and the indicated ErbB1 mutant expression vectors. The resulting cell lysates were subjected to immunoprecipitation with anti-Myc antibodies and immunoblotted with monoclonal antibody directed to the extracellular portion of ErbB1 (anti-111). Total cell lysates are shown.

In our previous report we have demonstrated that nucleolin interacts with the four ErbB receptors and that this interaction is functional as it induces receptor activation in a ligand independent manner [28]. In order to identify the receptor domain responsible for the interaction with nucleolin several deletion mutants in the cytoplasmic tail of the ErbB1 receptor were generated. The scheme of the designed deletion mutants is presented in Figure 1A. The mutants were examined for their ability to bind nucleolin by co-immunoprecipitation. As shown in Figure 1B, only two mutants failed to be precipitated by nucleolin; ΔCyt 1 and ΔNLS. These results suggest that the region in ErbB1, important for nucleolin binding, may be the ErbB1 nuclear localization sequence (NLS) (RRRHIVRKRTLRR (amino acids 669–681)). This region is known to regulate ErbB1 activity since it mediates the nuclear localization of EGFR [33] as well as receptor kinase activation [32].

**Figure 2 pone-0006128-g002:**
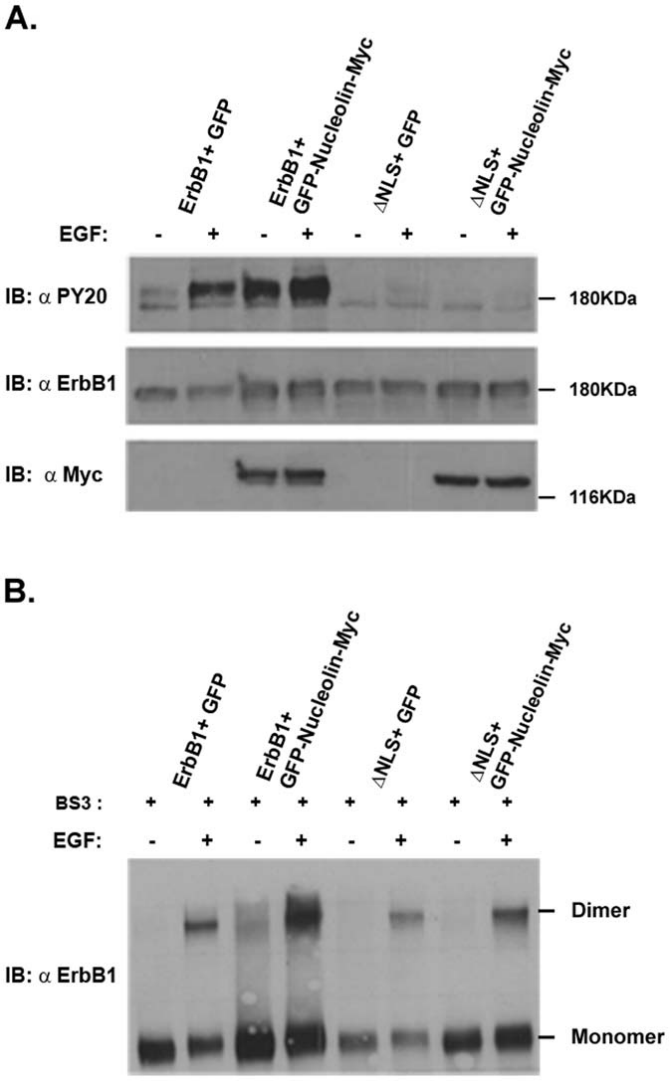
The ΔNLS mutant of ErbB1 is not activated in the presence of nucleolin or in the presence of its ligand, EGF. HEK-293T cells were co-transfected with pEGFP-nucleolin-Myc and either the wild type ErbB1 or ΔNLS ErbB1 deletion mutant expression vectors. Following 20 minutes serum deprivation, cells were treated with or without EGF (100 ng/ml) for 10 minutes. (A) Cell lysates were immunoblotted with monoclonal antibody anti PY20. As control cell lysates were immunoblotted with polyclonal antibody to ErbB1, or monoclonal antibody anti Myc as indicated. (B) Cell lysates were incubated with BS3 cross linker and immunobloted with polyclonal antibody anti ErbB1.

**Figure 3 pone-0006128-g003:**
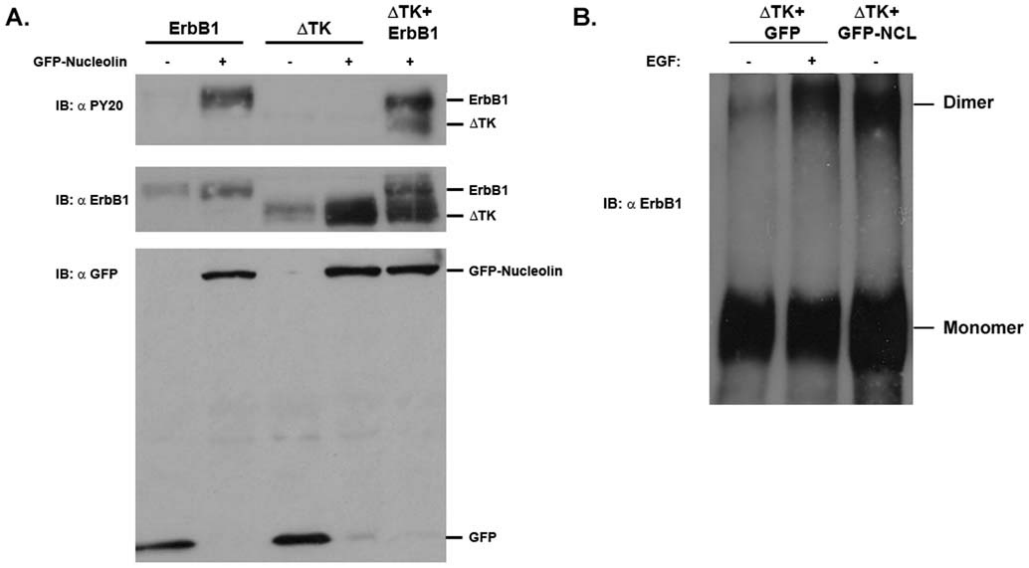
The ΔTK mutant of ErbB1 does not undergo nucleolin-mediated activation unless wild type ErbB1 is present. HEK-293T cells were co-transfected with pEGFP-nucleolin-myc and the wild type ErbB1 or ΔTK ErbB1 deletion mutant or both expression vectors. Following 20 minutes starvation cells were treated with EGF (100 ng/ml) for 10 minutes. (A) Cell lysates were immunoblotted with monoclonal antibody anti PY20. As control cell lysates were immunoblotted with polyclonal antibody to ErbB1, or monoclonal antibody anti Myc as indicated. (B) Cell lysates were incubated with BS3 cross linker and immunoblotted with polyclonal antibody to ErbB1.

Since the ΔCyt mutants lack phosphorylated tyrosine residues in the cytoplasmic tail of the receptor they are not functional in mediating signals. Nevertheless, it was important to examine whether nucleolin can affect the ΔNLS mutant-mediated signals and whether the ΔTK mutant that contains the cytoplasmic tail (phosphorylation sites) and binds nucleolin can transmit signals following co- expression. To determine whether nucleolin affects ErbB1 or ErbB1 ΔNLS mutant tyrosine phosphorylation we co-expressed ErbB1 or ErbB1 ΔNLS mutant and nucleolin in HEK-293T cells. Indeed, when nucleolin was co-expressed with ErbB1 it induced receptor phosphorylation even in the absence of EGF. However, when ErbB1 ΔNLS mutant was co-expressed with nucleolin no phosphorylation was detected even in the presence of EGF (Figure 2A). Cross linking experiments confirm that EGF but not nucleolin can induce dimerization of the ErbB1 ΔNLS receptor mutant (Figure 2B). This result correlates with the finding that ErbB1 ΔNLS mutant cannot interact with nucleolin. Similarly, the ErbB1 mutant in the tyrosine kinase domain (ΔTK) failed to undergo phosphorylation, when co-expressed with nucleolin (Figure 3A). When wild type ErbB1 was co-expressed with nucleolin and ΔTK, nucleolin induced ErbB1 and ErbB1 ΔTK mutant phosphorylation. The ΔTK although binds nucleolin, do not undergo nucleolin-associated phosphorylation unless wild type ErbB1 is present (Figure 3A). Nucleolin however, induces dimerization of the ΔTK as evident by the crosslinking experiments (Figure 3B). Thus, nucleolin-induced ErbB1 phosphorylation depends on receptor dimerization and functional receptor tyrosine kinase domain.

**Figure 4:**
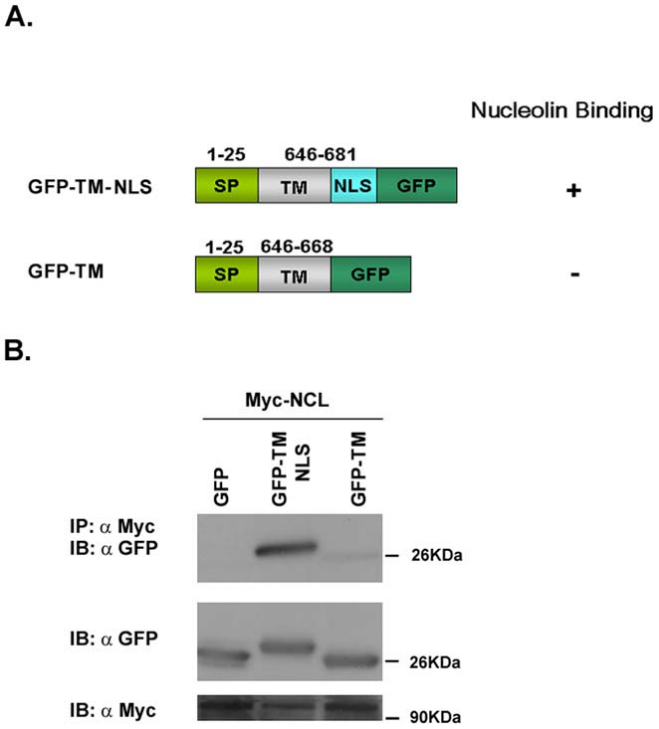
The NLS of ErbB1 is important for ErbB1 nucleolin interaction. (A) Schematic presentation of the chimeric protein expression vectors. (B) Cells were transfected with nucleolin-myc and either pEGFP (empty vector), pEGFP-TM-NLS or pEGFP-TM expression vectors. The resulting cell lysates were subjected to fractionation and the pellets were subjected to immunoprecipitation with anti-Myc antibodies and immunoblotted with anti GFP antibodies. As control, total cell lysates were blotted with anti-GFP and anti-Myc antibodies.

To further study the importance of the receptor NLS region for nucleolin interaction new expression vectors of chimeric proteins were generated as shown in the scheme (Figure 4A). The chimeric proteins were examined for their ability to bind nucleolin by co-immunoprecipitation. As shown in Figure 4B, only the chimeric protein that contains the NLS was precipitated by nucleolin. The chimeric protein that contained only the transmembrane domain failed to be precipitated by nucleolin. These results further support the importance of the NLS region of ErbB1 for nucleolin interaction.

### Analysis of nucleolin domains responsible for the interaction with ErbB receptors

**Figure 5:**
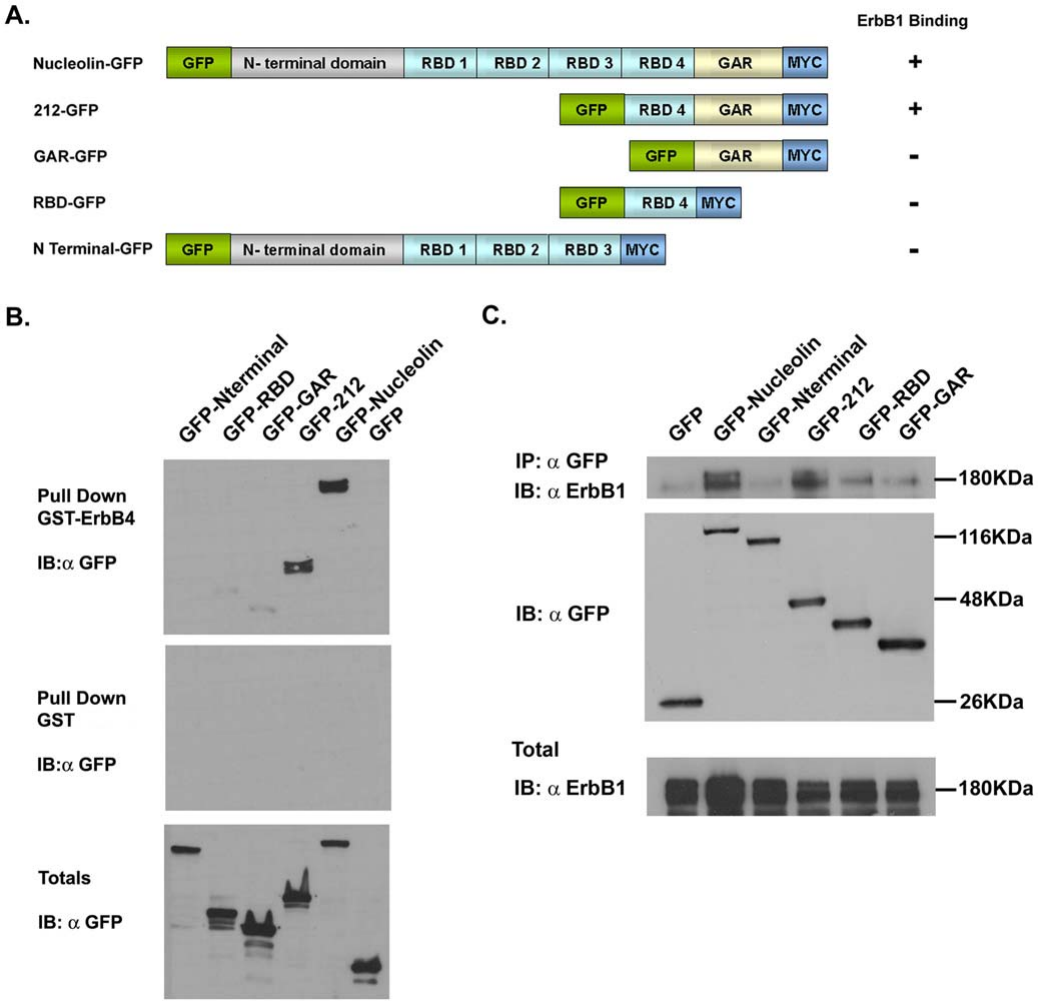
The 212 C-terminal aa of nucleolin are important for nucleolin ErbB interaction. GST pull-down assay showing ErbB4 interaction with nucleolin and nucleolin mutants. (A) Schematic presentation of nucleolin deletion mutants. (B) HEK-293T cells were transfected with pEGFP (empty vector), pEGFP-nucleolin-myc or pEGFP-nucleolin-myc mutant expression vectors. The resulting cell lysates were incubated with immobilized GST-ErbB4 or GST. Proteins retained on the beads were resolved by SDS-PAGE and then processed for Western blot using a monoclonal antibody anti- GFP. (C) The resulting cell lysates were subjected to fractionation and the pellets were subjected to immunoprecipitation with anti-GFP antibody and immunoblotted with anti ErbB1 polyclonal antibody.

Previously we have demonstrated that Glutathione-S-transferase-ErbB4 (GST-ErbB4) pulled down nucleolin. Further analysis has shown that nucleolin interacts and activates the four ErbB receptors in a ligand independent manner [28]. To further investigate the interactions between ErbB and nucleolin we used the pull down assay. A chimeric fusion protein of GST and ErbB4 cytoplasmic tail (GST-ErbB4) was used as a bait to pull down nucleolin mutants. Several deletion mutants of nucleolin were generated. The scheme of the designed deletion mutants is presented in Figure 5A. Cell extracts were prepared from HEK-293T cells transfected with the expression vectors encoding the wild type and the various nucleolin mutants N-ter, 212, RBD, GAR, all fused to GFP. As shown in Figure 5B, GST-ErbB4 pulled down wild type nucleolin and the 212 C-terminal amino acids mutant but not the N-terminal mutant. Moreover the GST-ErbB4 pulled down significantly less effectively, both the RBD and the GAR mutants, which are part of the 212 C-terminal portion of nucleolin. The results indicate that the C-terminal region of nucleolin is important for its binding to the cytoplasmic tail of ErbB4 receptor. To further examine the interaction between ErbB1 and nucleolin we have used co immunoprecipitation experiments on HEK-293 cells stably over expressing ErbB1 receptor transfected with the various nucleolin mutant expression vectors. As shown in Figure 5C, ErbB1 was precipitated effectively by both full-length nucleolin and 212 C-terminal amino acids mutant, less effectively to GAR and RBD mutants, but not by the N-terminal mutant of nucleolin. These results further support the importance of this nucleolin region (212 mutant) for the interaction with ErbB1 receptors.

**Figure 6:**
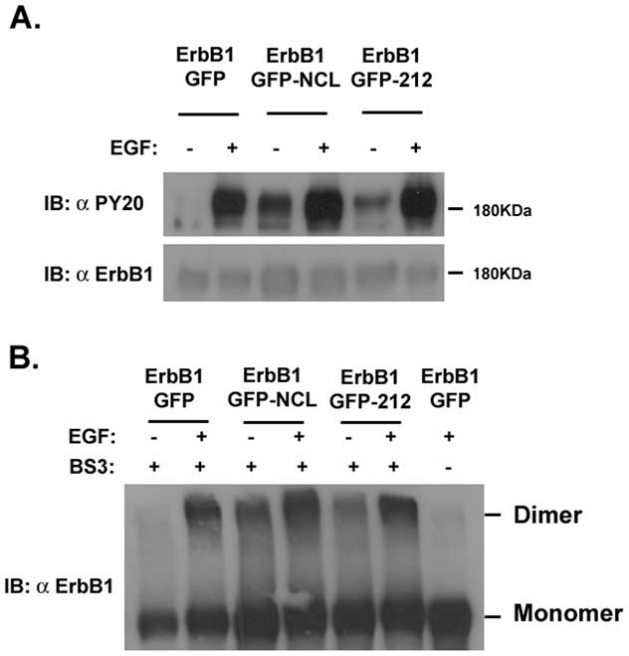
The 212 C-terminal aa of nucleolin are sufficient to induce ErbB1 phosphorylation and dimerization. (A) Cells were transiently co-transfected with expression vector of ErbB1 receptor and with either pEGFP, pEGFP-nucleolin-Myc (GFP-NCL) or pEGFP-212-nucleolin-Myc (GFP-212) mutants. Following 20 minutes serum deprivation, cells were treated with or without EGF 100 ng/ml for 10 minutes. Cell lysates were immunobloted with monoclonal antibody PY20. As a control lysates were immunoblotted with polyclonal antibody anti ErbB1 as indicated. (B) Cells were transiently co-transfected with expression vector of ErbB1 receptor and with either pEGFP, pEGFP-nucleolin-Myc (GFP-NCL) or pEGFP-212-nucleolin-myc (GFP-212) mutants. Following 20 minutes serum deprivation, cells were treated with or without EGF 100 ng/ml for 10 minutes. Cell lysates were incubated with BS3 cross linker and immunobloted with polyclonal antibody anti ErbB1.

To determine whether the 212 C-terminal amino acids mutant of nucleolin affects ErbB1 tyrosine phosphorylation, we co-expressed ErbB1 protein with or without nucleolin or the 212 C-terminal amino acids mutant, in HEK-293 cells. Both nucleolin and 212 C-terminal amino acids mutant of nucleolin when co-expressed with ErbB1 induced receptor phosphorylation even in the absence of EGF. This was evident using anti-phosphotyrosine antibodies that recognize phosphorylated tyrosines (Figure 6A). As control for receptor phosphorylation, cells were stimulated with EGF as indicated. Expression of nucleolin N-ter, RBD and GAR mutants with ErbB1 failed to induce receptor phosphoryaltion (data not shown). These results suggest that the 212 C-terminal amino acids of nucleolin are sufficient to induce ErbB1 phosphorylation. To address the possibility that the 212 C-terminal amino acids of nucleolin induces the competence of ErbB1 dimerization and trans-auto-phosphorylation, we examined the effect of the 212 C-terminal amino acids of nucleolin on receptor dimerization, which was determined by covalent crosslinking (Figure 6B). Clearly, ligand-independent formation of ErbB1 dimers is potentiated following the expression of nucleolin and 212 C-terminal amino acids of nucleolin. As a control homodimerization of the ErbB1 receptors was induced by the ligand EGF. These observations may suggest that the receptor phosphorylation induced by 212 C-terminal amino acids of nucleolin may result due to receptor dimerization.

### Analysis of ErbB1 and nucleolin domains localization and anchorage-independent growth in HEK-293 over expressing cells

**Figure 7:**
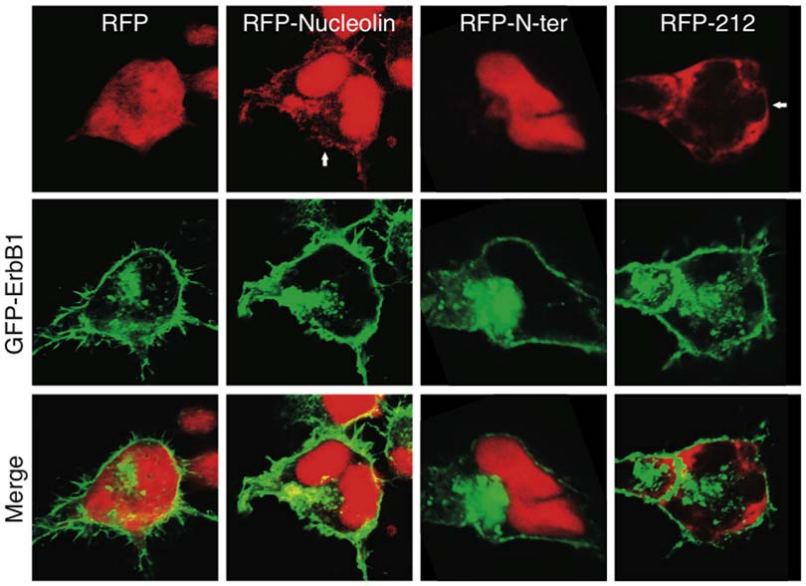
Cellular distribution of ErbB1 and nucleolin mutants. HEK-293T cells were transfected with EGFP-ErbB1 and either pDsRed, pDsRed -nucleolin, pDsRed -N-ter or pDsRed -212-C-ter. 48 h following transfection cells were fixed and subjected to confocal microscopy analysis. GFP-ErbB1 is visualized in green and pDsRed expression vectors are seen in red. Representative cells are presented.

Since the 212 C-terminal amino acid of nucleolin mediated similar effects as the full-length protein we next examined the localization of these two proteins. HEK-293T cells were transiently co-transfected with GFP-ErbB1 and either pDsRed empty vector, pDsRed-nucleolin, pDsRed-N-ter or pDsRed-212 C-ter, their sub cellular localization was studied 48 hours later by confocal microscopy. As exemplified in Figure 7 and in agreement with previous reports [34], GFP-ErbB1 was localized to the plasma membrane and to the cytoplasm vesicular compartment. Full-length nucleolin was mainly localized to the nucleus as well as to the plasma membrane. GFP-ErbB1 and pDsRed-nucleulin co-localized to the same membranous compartments. GFP-ErbB1 and DsRed-N-ter showed no co-localization since DsRed-N-ter was localized exclusively to the nucleus whereas ErbB1 was excluded from the same compartment. Interestingly, DsRed-212-C-ter co-localized with GFP-ErbB1 at the cell surface. These results are in agreement with the above-described interactions and further support the notion that nucleolin 212 C-terminal amino acids are sufficient for the interaction with ErbB1 on the cell surface.

**Figure 8:**
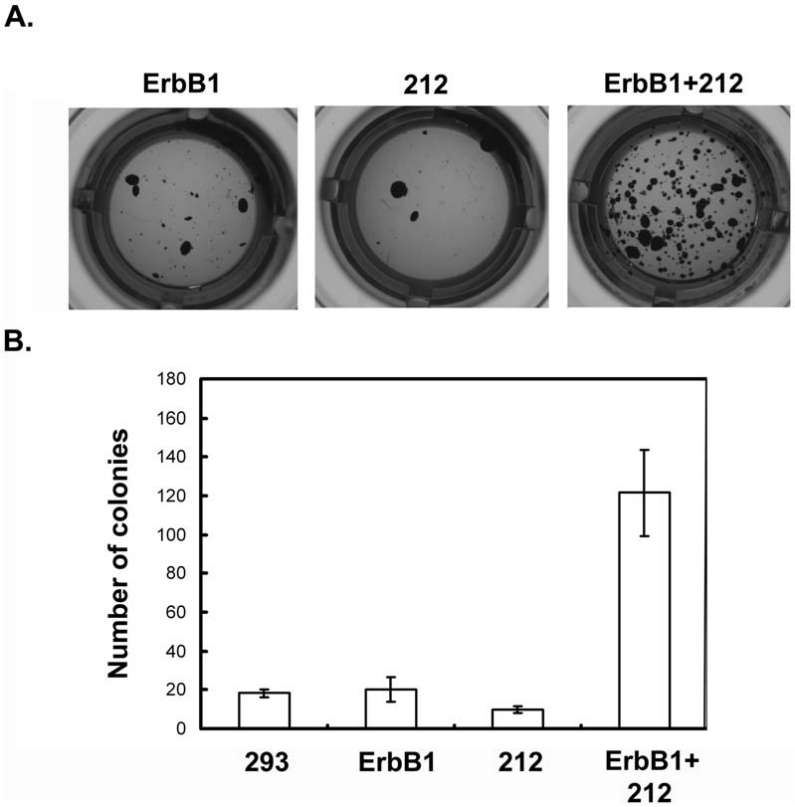
Nucleolin-212-C-ter mutant and ErbB1- induced anchorage-independent growth. (A) HEK-293 cells stably expressing empty vector (not shown), ErbB1, nucleolin-212-C-ter or ErbB1 and nucleolin-212-C-ter were seeded in soft agar (4000 cells/well in 96 well plates) in medium containing 10% FBS, 0.3% agar, in the presence of 100 ng/ml EGF. The extent colony formation was determined 2 weeks later. Cells were dyed with MTT and the wells were photographed and colonies were counted. Representative image is presented. (B) Results were quantified using image analyzer program Image pro-Plus. The results are presented as the number of colonies at size larger then 0.01 mm^2^. Note that nucleolin-212-C-ter+ErbB1 but not each protein alone induced significantly more colonies (p<0.001). Results are the mean ± SD 6 determinations from each clone (3 clones from each stable cell line). Each experiment was repeated twice with similar results.

In order to further examine the effect of 212 C-terminal amino acids of nucleolin on ErbB1-mediated responses we prepared HEK-293 cells stably expressing empty vector, ErbB1, 212-C-ter or ErbB1 together with 212-C-ter. In order to assess effects of ErbB1 and 212-C-ter on colony formation, HEK-293 stable clones were plated in soft agar and maintained in culture for 14 days. The number and size of colonies were then estimated from three individual clones. Results of a typical experiment are shown in Figure 8. HEK-293 cells overexpressing either ErbB1 or 212-C-ter, formed relatively low number of colonies. However, HEK-293 cells overexpressing ErbB1 and 212-C-ter, formed relatively significantly high number of colonies in soft agar (p<0.001). Pools of mock-transfected HEK-293 cells were plated in soft agar as a control and showed relatively low number of colonies in soft agar (Figure 8B). These results indicate that the 212-C-ter interaction with ErbB 1 is functional in mediating the anchorage-independent growth of the cells overexpressing both ErbB1 and 212-C-ter.

## Discussion

The ErbB receptors are activated by their cognate growth factors under normal physiological conditions [14], [35]. Ligand binding to ErbB receptors induces the formation of receptor homo- and hetero-dimers and activation of the intrinsic kinase domain, resulting in phosphorylation on specific tyrosine residues within the cytoplasmic tail. These phosphorylated residues serve as docking sites for a range of proteins, the recruitment of which leads to the activation of intracellular signaling pathways [13]. Recently, we have demonstrated that cell-surface ErbB receptors, interact with nucleolin via their cytoplasmic tail [28]. Nucleolin is a ubiquitous, nonhistone, nucleolar, multifunctional phosphoprotein that is also overexpressed in cancer cells [16], [36]. It was demonstrated that nucleolin not only interacts with ErbB proteins but may also affect their activation [28]. Overexpression of ErbB1 and nucleolin may lead to receptor dimerization, phosphorylation and to anchorage independent growth [28]. Based on these results we have previously suggested that nucleolin may modulate ErbB receptors activities.

In the present study, we characterized the protein domains responsible for this interaction and for receptor activation. We found that the interaction between nucleolin and ErbB1 depends on the NLS domain of ErbB1 (RRRHIVRKRTLRR (amino acids 669–681)) (Figures 1–4). This region is known to regulate ErbB1 activity, since it mediates the nuclear localization of EGFR [33] as well as receptor kinase activation [32]. However, other studies demonstrated that mutation of EGFR tripartite NLS or deletion of the ErbB2 tripartite NLS did not affect receptor protein membrane localization and activation of MAPK signaling [37]. In our study we demonstrated that upon nucleolin or ligand stimulation the DNLS mutant of ErbB1 does not undergo phosphorylation (Figure 4A). However, further examination of the receptor dimerization using BS3 crosslinker, demonstrated that EGF can induce DNLS mutant dimerization. Studies have shown that upon ligand binding the dimerization process starts from the extracellular region of EGFR [38]. Hence, by using the cross linker we observed receptor dimerization, although the dimerization/activation process was not completed, as demonstrated by the lack of phosphorylation (Figure 4). Thus, EGF but not nucleolin can induce the dimerization of DNLS mutant. These results support our findings that the DNLS ErbB1 mutant does not interact with nucleolin. In addition, using expression vectors of TM-NLS compared to TM mutants further demonstrate the importance of the NLS for nucleolin interaction. Interestingly, the NLS region of ErbB1 and ErbB4 are highly conserved (RRKSIKKKRALRR amino acids 666–678 in ErbB4) which may account for the similar interaction with nucleolin. Accordingly, our present results strongly suggest that the NLS region of the receptor is the nucleolin-binding domain. Using the ΔTK ErbB1 mutant we demonstrated that the tyrosine kinase domain, although not essential for ErbB1/nucleolin interaction, is crucial for ErbB1 activation induced by nucleolin. Our results further indicate that ΔTK undergoes EGF or nucleolin induced dimerization but not phosphorylation indicating that nucleolin-mediated receptor activation depends on the receptor kinase domain activity (Figure 3).

Further examination of the nucleolin domains responsible for this interaction and for receptor activation was performed. We found that the C-terminal 212 amino acids of nucleolin (Nuc-212) are necessary for the interaction between nucleolin and ErbB4 or ErbB1 (Figure 5). We have also demonstrated that the last 212 amino acids of nucleolin are sufficient for the activation of ErbB1 receptor (Figure 5). These amino acids are comprised of two distinct regions, RBD and GAR. Neither RBD nor GAR effectively interacted with either ErbB4 or ErbB1 (Figure 5), ergo the entire sequence is important for the stable interaction with ErbB receptors. A specific interaction of (Nuc-212) with the Mo-MuLV Gag precursor was previously demonstrated. This binding of (Nuc-212) resulted in a strong inhibition of virion assembly or release in vivo [39]. However, 212 C-ter nucleolin (Nuc-212) binds and activates ErbB1 in a similar manner as the full-length nucleolin thus, indicating that this region is sufficient for binding and activation.

Nucleolin contains three main structural domains: N-terminal region containing several long stretches of acidic residues; central globular domain containing four RNA binding sites; C-terminal domain known as arginine-glycine rich domain (GAR domain). As previously described the 212-C-ter mutant we used is comprised of the fourth RBD and the GAR domains. Upon stimulation of cell proliferation, cytoplasmic nucleolin is translocated to the cell surface [40]. Cell surface nucleolin serves as a low affinity receptor for HIV-1 and binds various growth factors via interaction with its GAR domain (i.e. midkine, pleiotrophin (PTN) and lactoferrin) [22], [41]–[43]. The GAR domain was further demonstrated to be the binding site of HB-19 pseudopeptide which was demonstrated to block nucleolin function as a low affinity receptor on the cell surface [41], [42]. Cell surface nucleolin could shuttle ligands between the cell surface and the nucleus thus acting as a mediator for the extracellular regulation of nuclear events [22], [23], [44]. In addition, accumulating data suggest that nucleolin binds other proteins such as the glucocorticoid receptor [45] and protein tyrosine phosphatase-sigma [46] and affects their activities. In the present study we demonstrate that the 212 C-terminal amino acids of nucleolin are sufficient to induce ErbB1 phosphorylation and dimerization (Figure 6). Importantly, it is demonstrated that ErbB1 and 212-C-ter nucleolin co-localize on the cell surface (Figure 7). Moreover this interaction is functional as it is sufficient to increase growth in soft agar (Figure 8). Taken together our results, we suggest that the 212 C-terminal amino acids of nucleolin bind to the NLS of ErbB1 on the cell surface. The interaction induces dimerization of ErbB1, resulting in phosphorylation through the tyrosine kinase domain.

There are increasing evidences that nucleolin expression on the cell surface is implicated in growth of tumor cells. Inhibition of nucleolin activity results in cell growth inhibition [24], [25]. It was demonstrated in several studies that AS1411, a quadruplex-forming oligonucleotide aptamer, targets nucleolin and inhibits cancer cells growth [26], [27]. In addition, ErbB receptor tyrosine kinases are major contributors to malignant transformation and they are frequently overexpressed in a variety of human carcinomas [47]. Thus, our results may suggest that the significance of nucleolin expression on the cell surface of tumor cell lines is to increase receptor-mediated activities.

## Materials and Methods

### Materials and Buffers

Human recombinant EGF was purchased from Boehringer Mannheim. Polyclonal rabbit anti- ErbB1 antibodies, monoclonal mouse anti-phosphotyrosine (PY20), mouse anti-GFP (B-2) were purchased from Santa Cruz Biotechnology (Santa Cruz, CA, USA). Polyclonal rabbit anti phosphorylated ErbB1 (Tyr1068) were purchased from Cell Signaling technology. Monoclonal mouse anti N' terminal EGFR (Ab 10) were from NeoMarkers (Lab Vision corporation). Monoclonal mouse anti c-Myc (9E10) was donated by Dr. Altchuler Yoram the Hebrew University, Israel. All other reagents were from Sigma. HNTG buffer contained 20 mM HEPES (pH 7.5), 150 mM NaCl, 0.1% Triton X-100 and 10% glycerol. Solubilization buffer contained 50 mM HEPES (pH 7.5), 150 mM NaCl, 1% Triton X-100, 1 mM EGTA, 1 mM EDTA, 1.5 mM MgCl_2_, 10% glycerol, 2 mM sodium vanadate, 1 mM phenylmethylsulfonylfluoride, 10 mg/ml aprotanin and 10 mg/ml leupeptin. Pull Down buffer contained 50 mM TRIS-HCl (pH 7.6), 20 mM MgCl2, 150 mM NaCl, 1 mM DTT, 2 mM sodium vanadate, 1 µM phenylmethylsulfonylfluoride, 10 µg/ml aprotanin, 10 µg/ml leupeptin, 0.5% NP40, 5 µg/ml pepstatine and 1 mM Benzamidine. Binding buffer contained 50 mM TRIS-HCl (pH 7.6), 10 mM MgCl_2_, 100 mM NaCl, 0.5 mM DTT and 0.5 mg/ml BSA.

### Cell lines

HEK-293, HEK-293T cell lines were grown in Dulbecco's modified Eagle's (DMEM) supplemented with 10% fetal bovine serum. Cells were transfected using jetPEI (Poly plus transfection, USA) or using calcium phosphate precipitation. Cell lysates were prepared 48 hours following transfection as described. The HEK-293 cells were used for stably expressing the ErbB-1 receptor and nucleolin c-terminal 212 amino acids (212). Expression vector pEGFP-ErbB-1 containing the coding region of ErbB-1 and expression vector pcDNA3-Myc-212 containing the 212 c-terminal region of nucleolin were introduced by CaPO4 transfection into HEK-293 cells either alone or together. The neomycin (G418) resistant colonies were checked for ErbB-1 or nucleolin-212 expression and several colonies (at least 4 from each combination) were selected for further analysis. Expression vector pcDNA3-ErbB-1 containing the coding region of ErbB-1 was introduced by CaPO4 transfection into HEK-293 cells. The neomycin (G418) resistant colonies were examined for ErbB-1 expression.

### DNA constructs

A C' terminally myc-tagged mouse nucleolin (pMT21-myc-nucleolin) was generously provided by Dr. Bacharach E. (Tel-Aviv University, Israel). Nucleolin-myc cDNA was amplified using the following primers: 5′ CCGGAATTCGCCACCATGGTGAAGCTCGCG and 5′CGAACCATGATGGCTCGAATCAC. The amplified fragment was digested with EcoRI and XbaI and cloned into pcDNA3 expression vector. Next we generated several nucleolin mutants using PCR amplification, digestion EcoRI and EcoRV and cloning into EcoRI and EcoRV digested pcDNA3-nucleolin-myc expression vector. N' terminal primers were: 5′CGGAATTCGCCACCATGGTGAAGCTCGCG and 5′GATATCGTTGCACTTAGG. 212 fragment (the C-terminal 212 amino acids of nucleolin) primers were: 5′CCGGAATTCGCCACCATGACAAAAGAACTC and 5′CGAACCATGATGGCTCGAATCAC. RNA binding domain 4 (RBD) primers were: 5′CCGGAATTCGCCACCATGACAAAAGAACTC and 5′GATATCCCCTTAGGTTGG. GAR primers were: 5′ CCGGAATTCGCCACCATGGGRGAAGGGTG and 5′GGAACCATGATGGCTCGAATCAC. In order to generate GFP-fused nucleolin-myc or nucleolin mutants-myc expression vectors, the pcDNA3-nucleolin-myc vector or pcDNA3-nucleolin-myc mutants were digested with EcoRI and ApaI and cloned into pEGFP-C2 and DsRed-C2 expression vectors.

The following ErbB1 mutants were generated: Δcyt1 fragment (1–667 aa) was amplified using PCR, digested with BstEII and XhoI and cloned into pcDNA3-ErbB1. Δcyt1 primers were: 5′TCGGGGAGCAGCGATGCGAC and

5′CCGCTCGAGCTATTACATGAAGAGGCCGATCCC. Δcyt2 fragment (1–1076 aa) was amplified using PCR, digested with BstEII and XhoI and cloned into pcDNA3-ErbB1. Δcyt2 primers were:5′ TCGGGGAGCAGCGATGCGAC and

5′ CCGCTCGAGTTACTATGAGCTGTATCGCTGCAAGAAG. Δcyt3 fragment (1–984 aa) was amplified using PCR, digested with BstEII and XhoI and cloned into pcDNA3-ErbB1. Δcyt3 primers were: 5′ TCGGGGAGCAGCGATGCGAC and 5′ CCCTCGAGTTACTAAAGGTAGCGCTGGGGGTCTCGG. In order to delete the tyrosine kinase domain (712–968 aa) ΔTK, pcDNA3-ErbB1 was digested with EcoRI. The EcoRI fragment encoding amino acids 1–711 and 969–1211 were cloned in pCDNA3 expression vector.

In order to delete the Nuclear Localization Signal (669–681 aa), ΔNLS fragment was amplified using 2 sets of primers: 5′ TGGGTACCCTGCTGCAGGAGAGGGAGC, 5′ CTCCGTGGTCATGCTCCAAT and

5′ AATACGACTCACTATAG, 5′ ACGGTACCCATGAAGAGGCCGATGCCAG.

The 2 fragments were digested with KpnI and BstEII or EcoRV respectively. The two fragments were cloned into pcDNA3-ErbB1 digested with BstEII and EcoRV.

GFP-TM-NLS mutant, which contains the signal peptide (aa 1–25), the trans membrane and the NLS domains (aa 646–681) was amplified using 2 sets of primers: 5′GAAGATCTGCCACCACCATGCGACCCTCCG, 5′CAGTGGCGATCAGAGCCCGACTCG and

5′ GGGCTCTGATCGCCACTGGGATG, 5′GGAATTCCCTCCGCAGCGTGC.

The 2 PCR products were used as template for a third amplification using the following primers: 5′GAAGATCTGCCACCACCATGCGACCCTCCG and 5′GGAATTCCCTCCGCAGCGTGC. The PCR product was digested with EcoRI and BglII and cloned into GFP-N2 expression vector digested with BstEII and EcoRV.

GFP-TM mutant, which contains the signal peptide (aa 1–25), and the trans membrane domain (aa 646–668) was amplified using 2 sets of primers: 5′GAAGATCTGCCACCACCATGCGACCCTCCG, 5′ CAGTGGCGATCAGAGCCCGACTCG and

5′ GGGCTCTGATCGCCACTGGGATG, 5′ GGAATTCCATGAAGAGGCCG.

The 2 PCR products were used as template for a third amplification using the following primers: 5′GAAGATCTGCCACCACCATGCGACCCTCCG and 5′GGAATTCCATGAAGAGGCCG. The PCR product was digested with EcoRI and BglII and cloned into GFP-N2 expression vector digested with BstEII and EcoRV.

### Lysate Preparation, Immunoprecipitation and Immunoblotting

Cells were exposed to the indicated stimuli. After treatment, cells were solubilized in lysis buffer. Lysates were cleared by centrifugation. For direct electrophoretic analysis, boiling gel sample buffer was added to cell lysates For other experiments, antibodies were first coupled to anti-mouse IgG agarose (for monoclonal antibodies) or protein A-sepharose (for polyclonal antibodies) for 1 h at RT. Then the proteins in the lysate supernatant were immunoprecipitated with aliquots of the beads-antibody complexes for 2 h at 4°C. The immunoprecipitates were washed three times with HNTG, resolved by SDS-polyacrylamide gel electrophoresis (PAGE) through 7.5% gels and electrophoretically transferred to nitrocellulose membrane. Membranes were blocked for 1 h in TBST buffer (0.02 M Tris-HCl pH 7.5, 0.15 M NaCl, and 0.05% Tween 20) containing 6% milk, blotted with 1 µg/ml primary antibodies for 2 h, followed by 0.5 µg/ml secondary antibody linked to horseradish peroxidase. Immunoreactive bands were detected with the enhanced chemiluminescence reagent (Amersham Corp, Buckinghamshire, UK). For cell fractionation, cells were homogenized, and the supernatant (soluble proteins) and the pellet (insoluble proteins) fractions were obtained by centrifugation (100,000×g, 30 min, 4°C) [48]. Samples of the pellet fractions were subjected to immunoprecipitation.

### Cross-linking

Cross-linking experiments were performed by addition of 2 mM

bis (sulfosuccinimidyl) suberate (BS3), to the lysis buffer for 20 min on ice. The chemical crosslinking reaction was stopped by adding 50 mM Glycine and the samples were resolved by SDS-PAGE [15].

### GST Pull-Down Assay

To characterize proteins that interact with the cytoplasmic tail of ErbB4, a GST-ErbB4 column was generated by absorbing 20 ml of GST-ErbB4 -producing E. coli lysate (resulting from a 1-liter culture) to 1 ml of glutathione-Sepharose (Sigma). A similar column was prepared from GST-producing E. coli. PC12 cell lysate was prepared and a total of 2 mg protein was loaded on GST or GST-ErbB4 columns (in the presence of 0.5 ml binding buffer) for 2 h at 4°C. After the incubation the beads were washed three times with binding buffer. The GST and GST-ErbB4 bound proteins were eluted in boiling sample buffer and resolved by SDS-PAGE through 7.5% gels and electrophoretically transferred to nitrocellulose membrane for immunoblot analysis.

### Imaging

HEK-293T cells were plated on glass cover slips placed in 6 wells plates at a density of 2×10^5^ cells perwell for 24 h before transfection. Cells were transfected with GFP-ErbB1 and either DsRed, DsRed-NCL, DsRed-Nter or DsRed-212. 48 hours later, cells were fixed by 4% paraformaldehyde at room temperature for 30 min. Cells were examined by fluorescent microscopy at 63× magnification with Zeiss 510 confocal microscopy.

### Soft Agar Assay

Cells were seeded at a density of 4000 cells/well in 96 well plates in DMEM containing 10% FBS. The cells were mixed with 0.05 ml (per each well) of 0.33% noble agar, and the mixture was poured onto a layer of 0.05 ml 1% noble agar in DMEM containing 10% FBS. The upper layer of the agar was covered with 0.1 ml of medium. Assays were performed in at least six repeats. The number and sizes of the colonies were estimated on day 14, using a binocular and a light microscope with the image analyzer program Image pro-Plus. Results are the mean±SD 6 determinations of each of the clones that were used (3 stable clones from each transfection).
